# Animate and Inanimate Words Demonstrate Equivalent Retrieval Dynamics Despite the Occurrence of the Animacy Advantage

**DOI:** 10.3389/fpsyg.2021.661451

**Published:** 2021-06-03

**Authors:** Michael J. Serra

**Affiliations:** Department of Psychological Sciences, Texas Tech University, Lubbock, TX, United States

**Keywords:** adaptive memory, animacy effect, animacy advantage, free recall, retrieval dynamics, serial position, probability of first retrieval, retrieval contiguity

## Abstract

People demonstrate a memory advantage for animate (living) concepts over inanimate (nonliving) concepts in a variety of memory tasks, including free recall, but we do not know the mechanism(s) that produces this effect. We compared the retrieval dynamics (serial-position effects, probability of first recall, output order, categorical clustering, and recall contiguity) of animate and inanimate words in a typical free recall task to help elucidate this effect. Participants were more likely to recall animate than inanimate words, but we found few, if any, differences in retrieval dynamics by word type. The animacy advantage was obtained across serial position, including occurring in both the primacy and recency regions of the lists. Participants were equally likely to recall an animate or inanimate word first on the tests and did not prioritize recalling words of one type earlier in retrieval or demonstrate strong clustering by animacy at recall. Participants showed some greater contiguity of recall for inanimate words, but this outcome ran counter to the animacy effect. Together, the results suggest that the animacy advantage stems from increased item-specific memory strength for animate over inanimate words and is unlikely to stem from intentional or strategic differences in encoding or retrieval by word type, categorical strategies, or differences in temporal organization. Although the present results do not directly support or refute any current explanations for the animacy advantage, we suggest that measures of retrieval dynamics can help to inspire or constrain future accounts for this effect and can be incorporated into relevant hypothesis testing.

## Introduction

Across a variety of memory tasks, people often remember more animate (living) concepts than inanimate (nonliving) concepts: *the animacy advantage*. Most often, researchers examine this effect in the context of the free recall of word lists and have consistently found a recall advantage for animate (e.g., tiger; engineer) over inanimate (e.g., couch; violin) words (e.g., Nairne et al., 2013; Bonin et al., 2014, 2015; Li et al., 2016; Popp and Serra, 2016, 2018; Gelin et al., 2017, 2019; VanArsdall et al., 2017; Leding, 2018, 2019; Meinhardt et al., 2018). The advantage can also occur for recognition (Leding, 2020), nonwords given animate properties (VanArsdall et al., 2013), and word pairs (e.g., VanArsdall et al., 2015; DeYoung and Serra, 2021).

We do not know the mechanism(s) that underlies the animacy advantage, but researchers have discredited some potential candidates. The animacy advantage *does not* seem to occur because animate words are easier to categorize (Gelin et al., 2017; VanArsdall et al., 2017), more mentally arousing (Popp and Serra, 2018), more emotionally arousing (Meinhardt et al., 2018), or more threatening (Leding, 2019, 2020) or invoke greater encoding effort (Bonin et al., 2015; Leding, 2018) than inanimate words. At present, there is conflicting evidence whether animate concepts involve greater visual imagery (i.e., Bonin et al., 2015; Gelin et al., 2019) and conflicting evidence that animate items attract more attention during encoding (i.e., Bonin et al., 2015; Hagen et al., 2018; Bugaiska et al., 2019; Johnson, 2019; Leding, 2019, 2020). Growing evidence suggests the effect might stem from the greater richness of encoding for animate items (e.g., Mieth et al., 2019; Mah et al., 2020; Meinhardt et al., 2020).

The purpose of the present study was *not* to directly test any previous or new hypotheses for the animacy advantage in memory. Rather, we suggest that comparing the retrieval dynamics (e.g., serial-position effects, probability of first recall, output order, categorical clustering, recall contiguity) of animate vs. inanimate words can help to identify or rule out potential mechanisms for the effect. Most published studies of the animacy advantage in free recall do not report aspects of retrieval dynamics, besides overall recall performance. One exception is Bonin et al. (2015): they found some tendency for participants to recall animate words before inanimate words, and the animacy advantage occurred across serial position in their lists.

The effect of presentation order (*serial position*) on the free recall of a list of items is one of the oldest documented effects in cognitive psychology (e.g., Robinson and Brown, 1926; Jenkins and Dallenbach, 1927). Compared to words from the middle of the list, participants typically show better memory for words from the beginning (*the primacy effect*) and better memory for words from the end (*the recency effect*). Much empirical evidence indicates that these two effects are independent (e.g., Murdock, 1962). For example, discouraging rehearsal during learning only reduces the primacy effect (e.g., Marshall and Werder, 1972; Tan and Ward, 2000), whereas including a distracter task between the last item presented and the test only reduces the recency effect (e.g., Postman and Phillips, 1965; Tan and Ward, 2000). The classic explanation for this independence (e.g., Waugh and Norman, 1965; Glanzer and Cunitz, 1966; Atkinson and Shiffrin, 1968; Milner, 1974; Moscovitch, 1982) is that the primacy effect reflects recall from long-term memory while the recency effect reflects recall from working memory. Although the animacy advantage is typically characterized as an effect of long-term memory (specifically, episodic memory; Nairne et al., 2017; VanArsdall et al., 2017), it can also occur for very short word lists presumably recalled from working memory (cf. Daley et al., 2020). Researchers studying the animacy advantage often design their studies to *prevent* the primacy effect and recency effects from occurring. For example, Nairne et al. (2013) included buffer words at the start and end of their lists and a distracter task between encoding and recall to deter these effects. In the present experiment, we did not include any buffer words or a distracter task to allow the primacy and recency effects to occur so we could consider if the animacy advantage favors long-term or working memory. Accounts of the animacy advantage that rely on preferential processing for animate over inanimate items might predict a larger animacy effect earlier in the list than later in the list, as it would become more difficult to favor one subset of the words as more words are presented at a fixed rate. In contrast, categorical accounts might predict a greater animacy advantage later in the list than earlier in the list, as the animate and inanimate subsets of words become more apparent.

The first item that participants recall from a list is the basis for the measure known as the *probability of first recall* (Stefanidi et al., 2018). When participants are not required to recall items in the order studied, they most often recall the first-studied word first when the test is delayed (Howard and Kahana, 1999, 2002) and recall the last-studied word first when the test immediately follows study (Hogan, 1975; Laming, 1999). The first item that participants recall from each list can, therefore, serve as a measure of recency as well as memory strength. A related aspect of retrieval is the *output order* in which participants recall the words at test. Although some studies concluded that participants recall items with greater memory strength earlier (e.g., Deese and Kaufman, 1957; Postman and Phillips, 1965); others concluded that participants strategically prioritize recalling weaker items sooner to maximize overall recall (e.g., Battig and Slaybaugh, 1969; Brown and Thompson, 1971). For both measures, we might expect first recall to favor animate over inanimate words if they are associated with greater memory strength. If participants are employing a strategy to maximize recall, however, they might recall inanimate words earlier than animate words.

Independent of the output order, participants might show *categorical clustering* at recall (e.g., recalling items of the same type together), which might reflect a categorical encoding strategy and can aid recall for that category. VanArsdall et al. (2017) disconfirmed this possibility to be a major contributor to the occurrence of the animacy advantage in list recall, but we considered it here for completeness. Those authors concluded that the lack of clustering suggests that the animacy advantage stems more from item-specific processing than a categorical strategy.

The idea of *contiguity* in retrieval assumes that the recall of one item enhances the recall of another item that occurred in close temporal proximity (i.e., serial position) during encoding (Kahana, 1996). Contiguity can be demonstrated by plotting a conditional response probability curve (CRP; Kahana, 1996; Howard and Kahana, 1999, 2002; Healey et al., 2019). The lag-CRP is typically asymmetric, favoring recall at forward over backward lags but dropping quickly in both directions (Kahana, 1996; Howard and Kahana, 1999, 2002). As our participants expected memory tests, contiguity should occur (cf. Hintzman, 2016). More importantly, we might expect animate items to show greater contiguity than inanimate items, as animate items might involve greater temporal organization (cf. Gelin et al., 2017; but see Blunt and VanArsdall, 2021). Lack of a difference in contiguity might instead support the idea that the animacy advantage stems from item-specific processing.

## Materials and Methods

### Participants

In prior studies (e.g., Popp and Serra, 2016, 2018), we obtained rather large animacy effects (*η_p_*^2^ > 0.2). Various *a priori* power analyses, therefore, suggest small sample sizes: as few as 15 participants. Given that some of the present analyses only involved portions of the data and some participants have missing values for some measures, we oversampled considerably from that estimate by convenience, to 94 participants. These 94 participants were undergraduate college students enrolled in Introductory Psychology at Texas Tech University. They participated for class credit. Their mean age was 18.91 years old (*SD* = 1.37). Of the participants, 65 reported their gender as female, and 29 reported their gender as male. We did not exclude any participants from the analysis.

### Materials

The materials were 1,097 words (554 animate and 543 inanimate) from which a custom computer program created a random set of animate (e.g., astronaut and chicken) and inanimate (e.g., bicycle and doormat) words for each participant. Two independent raters identified the words as either animate or inanimate ahead of time; we only used words for which they agreed. The animate and inanimate words had a similar mean number of letters (7.42 vs. 7.20, respectively), valence (5.13 vs. 5.31) and arousal (4.41 vs. 3.99) ratings (Warriner et al., 2013), concreteness ratings (4.26 vs. 4.68; Brysbaert et al., 2014), and age of acquisition (8.24 vs. 7.68; Kuperman et al., 2012). We provide the words, their attributes, and the likelihood of recalling each in the present experiment at https://osf.io/rfbc2/. MANOVA revealed that the animate and inanimate words differed statistically on all but the number of letters (all other *ps* < 0.01), but each of these variables accounted for less than 1% of the variance in the likelihood of recall. Most of these differences would have favored the recall of inanimate words, yet we still obtained the animacy advantage (as in earlier experiments, with a *η*_p_^2^ around 0.2).

### Procedure

In each session, up to five participants completed the task on individual computers running a custom computer program, in the same room, separated by cubicle dividers. Task instructions indicated that the participants would study five lists of words and test over each list but did not mention animacy. Before beginning the task, the computer program randomly selected 50 animate and 50 inanimate words for each participant from the larger set. No word was used more than once per participant. From the 50 animate and 50 inanimate words, the program created five 20-word lists for each participant (each list did not necessarily have 10 animate and 10 inanimate words).

The participants then studied their first list. The computer program presented each word visually on the screen, one at a time, for 5 s each (with a blank, 250-millisecond inter-item interval). After presenting the last word in the list, the computer program requested that the participants type as many words as they could recall from the list into a field on the computer screen. The participants clicked on a “finished” icon when they could not recall any additional words. This procedure was then repeated for the other four lists. We did not include buffer items at the beginning or end of the lists or a distracter task between study and recall, to allow for the primacy and recency effects.

## Results

We provide the data from the present experiment at https://osf.io/rfbc2/.

### The Animacy Advantage

We scored recall as either correct or incorrect; we did not award partial credit. For words that the participants spelled incorrectly, we accepted them if they appeared to indicate the correct word phonetically (e.g., accepting “lepard” for “leopard”). We did not award extra points for words recalled more than once in the same list, nor did we penalize or award points for intrusions. We also recorded the output order for the correctly recalled words on each list (if recalled more than once, we used the first output position).

We first considered the main effect of animacy on free recall to ensure that the effect obtained across the five lists and to check for proactive interference (Table 1). Recall performance was greater for animate than inanimate words, *F*(1,93) = 21.68, *MSE* = 246.97, *p* < 0.001, *η_p_*^2^ = 0.19. Recall did not differ across lists, *F*(4,372) = 1.48, *MSE* = 291.37, *p* = 0.207, *η_p_*^2^ = 0.02. Animacy and list did not interact, *F*(4,372) = 1.11, *MSE* = 235.31, *p* = 0.352, *η_p_*^2^ = 0.01. We, therefore, collapsed the data on list number for the subsequent analyses.

**Table 1:** Percent recall and output order by animacy and list number.

	Percent recall				Output order			
	Inanimate		Animate		Inanimate		Animate	
List #	M	SD	M	SD	M	SD	M	SD
List 1	38.18	17.20	46.33	18.05	4.86	1.61	4.88	1.60
List 2	41.04	20.40	46.44	18.87	5.17	1.88	5.14	1.89
List 3	44.48	21.07	47.76	19.96	5.13	2.03	5.34	1.86
List 4	41.88	21.69	46.97	23.20	5.29	2.29	5.05	1.99
List 5	41.81	22.19	43.74	22.86	5.21	2.43	5.13	2.26

Percent recall is the mean percentage of words of each type that participants correctly recalled on each list. Output order is the mean output order for words of each type that participants correctly recalled on each list.

For completeness, we also considered intrusions. Participants averaged only 0.45 (*SD* = 0.70) *total* inanimate intrusions and 0.34 (*SD* = 0.61) *total* animate intrusions after summing across their lists. Intrusions did not differ by animacy, *F*(1,93) = 1.29, *MSE* = 0.41, *p* = 0.260, *η_p_*^2^ = 0.01. Given the very low rate of intrusions, we did not consider them further.

### Serial-Position Effects

**Figure 1:**
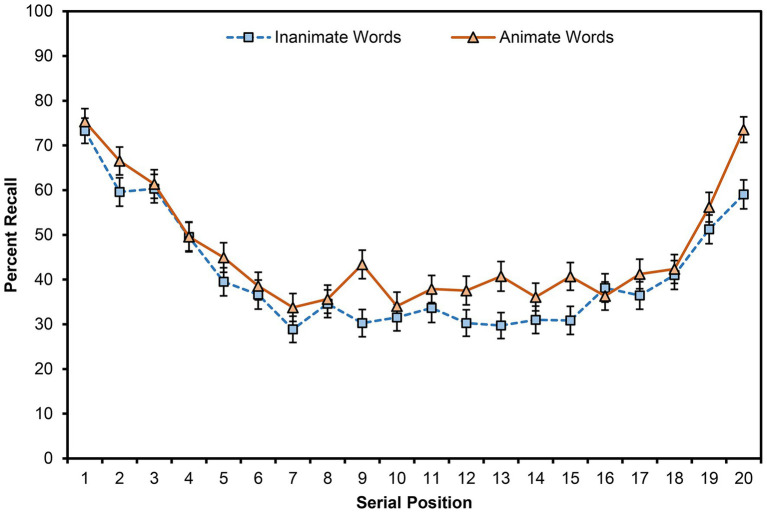
The mean free recall (percent recalled) for animate and inanimate words that were presented at each serial position. Results are collapsed on list number. Error bars are one SEM.

Figure 1 shows retrieval by animacy across serial position in the lists (collapsed on list number). Some researchers characterize the primacy region as the first three or four items in a list and the recency region as the last four (or more) items in a list (e.g., Stefanidi et al., 2018); others limit these regions to the first two and last two items in each list, respectively (e.g., Kelly and Risko, 2019).

First, we compared participants’ recall for animate and inanimate items that appeared in each fifth (quintile) of the lists (Table 2). Recall performance was greater for animate than inanimate words, *F*(1,93) = 24.60, *MSE* = 232.30, *p* < 0.001, *η_p_*^2^ = 0.21, and recall differed across quintiles, *F*(4,372) = 76.66, *MSE* = 355.56, *p* < 0.001, *η_p_*^2^ = 0.45. Contrasts revealed that recall was greater for items in the first quintile of the lists than for any other quintile (all *ps* < 0.001), indicating the primacy effect. Similarly, recall was greater for items in the fifth quintile of the lists than for items in the second, third, and fourth quintiles (all *ps* < 0.001), indicating the recency effect. Animacy and quintile did not interact, *F*(4,372) = 0.58, *MSE* = 202.59, *p* = 0.679, *η_p_*^2^ = 0.01.

**Table 2:** Percent recall and output order by animacy and item grouping.

	Percent recall				Output order			
	Inanimate		Animate		Inanimate		Animate	
Item grouping	M	SD	M	SD	M	SD	M	SD
**Even quintile split**								
Items 1–4	60.79	21.59	63.77	23.27	4.45	1.81	4.37	1.92
Items 5–8	35.05	21.94	38.60	21.36	6.57	2.05	6.15	1.88
Items 9–12	32.08	20.42	37.87	18.61	6.56	2.24	6.29	2.11
Items 13–16	32.13	18.89	38.78	21.99	6.85	2.48	6.60	2.45
Items 17–20	47.47	20.10	53.15	21.45	5.13	2.80	5.12	2.99
**Three region split**								
Items 1 & 2	67.17	25.16	71.69	26.63	4.13	2.25	3.75	2.12
Items 10 & 11	33.69	28.16	36.74	25.16	6.95	2.53	6.44	2.38
Items 19 & 20	56.96	28.51	63.83	28.17	4.57	3.22	5.04	3.37

Percent recall is the mean percentage of words of each type that participants correctly recalled in each specified range of the lists, collapsed across the five lists. Output order is the mean output order for words of each type that participants correctly recalled in each specified range of the lists, collapsed across the five lists.

Second, we compared participants’ recall for animate and inanimate items that appeared in the first two, middle two, and last two positions in the lists (Table 2). Recall performance was greater for animate than inanimate words, *F*(1,93) = 5.22, *MSE* = 626.27, *p* = 0.025, *η_p_*^2^ = 0.05, and recall differed by region, *F*(2,186) = 74.98, *MSE* = 788.09, *p* < 0.001, *η_p_*^2^ = 0.45. Contrasts revealed that recall was greater for items in the first region than for items in the middle (*p* < 0.001), indicating the primacy effect, and recall was greater for items in the last region than for items in the middle (*p* < 0.001), indicating the recency effect. Recall was also greater for items in the primacy region than for items in the recency region (*p* = 0.004). Animacy and region did not interact, *F*(2,186) = 0.38, *MSE* = 454.32, *p* = 0.682, *η_p_*^2^ < 0.01.

It seems that the animacy advantage is consistent across serial position in lists (cf. Bonin et al., 2015). This outcome suggests that the advantage does not favor either long-term memory or working memory and occurs for both memory systems (cf. Daley et al., 2020). This finding is inconsistent with accounts of the animacy advantage that rely on preferential rehearsal for animate over inanimate items (which might produce a larger animacy advantage earlier in the list) and with categorical-retrieval accounts (which might produce a larger animacy advantage later in the list).

### Probability of First Recall

We examined the first word that participants recalled on each test. There was a slight advantage for recalling an animate word (56% of first words recalled) vs. an inanimate word (44%), but this difference in proportions was not different from chance (*z* = 1.16, *p* = 0.245, 95% *CI* = 45.38 to 66.23%). Of all the words recalled first, 30.8% were the first word in that list and 28.9% were the last word in that list. Although the last item presented is most often the first word recalled when there is no distracter between study and recall (e.g., Howard and Kahana, 1999, 2002), that tendency did not occur here. A similar pattern emerged when considering animacy. When the first word recalled was inanimate, that word was studied first 33.7% of the time and last 28.3% of the time. When the first word recalled was animate, that word was studied first 28.6% of the time and last 29.4% of the time. These proportions did not differ by animacy, *χ*^2^ (1, 279) = 0.69, *p* = 0.406.

Participants were clearly more likely to recall animate than inanimate items (suggesting that the former have greater memory strength) and were most likely to recall the first-studied or last-studied word first on the tests (suggesting that memory strength predicted first recall). Any difference in memory strength by animacy, however, did not result in prioritized first recall for animate words (cf. Deese and Kaufman, 1957; Postman and Phillips, 1965; Stefanidi et al., 2018).

### Output Order

We calculated the mean output order for correctly recalled items. First, we considered output order by animacy across the five lists (Table 1). The output order did not differ by animacy, *F*(1,77) = 0.07, *MSE* = 1.97, *p* = 0.792, *η_p_*^2^ < 0.01, and did not differ across lists, *F*(4,308) = 1.16, *MSE* = 2.76, *p* = 0.329, *η_p_*^2^ = 0.02. Animacy and list did not interact, *F*(4,308) = 0.49, *MSE* = 2.12, *p* = 0.744, *η_p_*^2^ < 0.01.

Second, we considered the output order by each quintile of the lists, collapsed on list number (Table 2). The output order did not differ for animate and inanimate words, *F*(1,70) = 3.40, *MSE* = 2.26, *p* = 0.070, *η_p_*^2^ = 0.05. The output order differed by quintile, *F*(4,280) = 25.03, *MSE* = 5.63, *p* < 0.001, *η_p_*^2^ = 0.26. Contrasts revealed that the output order was lower for items in the first quintile than for any other quintiles (all *ps* < 0.001), except for the fifth quintile (*p* = 0.075). Similarly, the output order was lower for items in the fifth quintile than for items in the second, third, or fourth quintile (all *ps* < 0.001). Animacy and quintile did not interact, *F*(4,280) = 0.44, *MSE* = 2.21, *p* = 0.781, *η_p_*^2^ = 0.01.

Third, we considered the output order of items from the first two (primacy), middle two (control), and last two (recency) positions of the lists, collapsed on list number (Table 2). The output order did not differ for animate and inanimate words, *F*(1,55) = 0.62, *MSE* = 2.58, *p* = 0.435, *η_p_*^2^ = 0.01. The output order differed by region, *F*(2,110) = 22.15, *MSE* = 10.02, *p* < 0.001, *η_p_*^2^ = 0.29. Contrasts revealed that the output order was lower for items in the primacy region than for items in the middle (*p* < 0.001), and the output order was lower for items in the recency region than for items in the middle (*p* < 0.001). The output order did not differ for items in the primacy and recency regions (*p* = 0.105). Animacy and region did not interact, *F*(2,110) = 2.36, *MSE* = 3.38, *p* = 0.099, *η_p_*^2^ = 0.04.

Together, these results suggest that participants were not, purposely or not, likely to recall either animate or inanimate words sooner than later on the tests. Much like the first-recall data, these outcomes are consistent with the idea that participants recall items with greater memory strength earlier (e.g., Deese and Kaufman, 1957; Postman and Phillips, 1965) in terms of primacy and recency, but inconsistent with any similar expectation that they might recall animate items before inanimate items. Further, these outcomes are inconsistent with the idea that participants might have strategically recalled inanimate items before animate items to maximize overall recall (e.g., Battig and Slaybaugh, 1969; Brown and Thompson, 1971).

### Categorical Clustering

Ignoring priority, we considered whether participants tended to recall animate and inanimate items together during a recall, which suggests a categorical recall strategy, by calculating absolute Kendall Tau nonparametric correlations between animacy and the output order for each participant on each list. Across the five lists, the mean correlations were 0.23 (*SD* = 0.20), 0.28 (*SD* = 0.20), 0.26 (*SD* = 0.22), 0.28 (*SD* = 0.21), and 0.31 (*SD* = 0.25), respectively. Although all correlations were above zero (all *ps* < 0.00, all *Cohen’s d* > 1.19), the magnitudes only averaged around 0.27. This result suggests that the participants had some tendency to recall animate and inanimate items together, but it does not suggest a strong categorical recall strategy (cf. VanArsdall et al., 2017).

### Recall Contiguity

**Figure 2:**
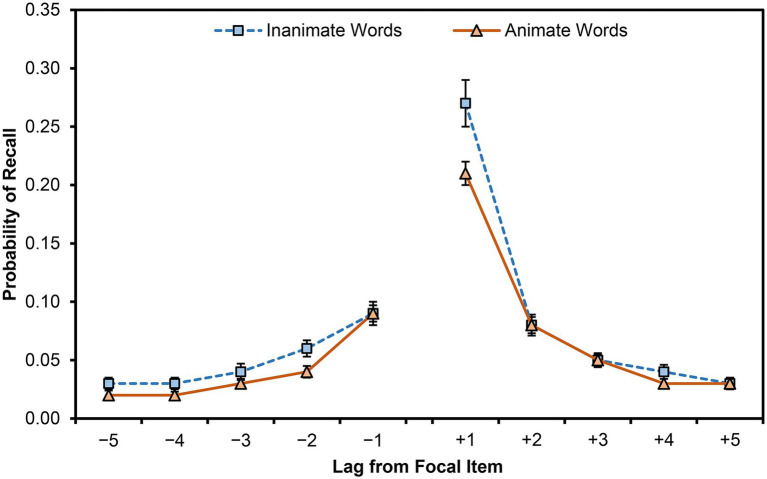
The mean lag conditional response probability (lag-CRP), within ±5 lag positions, split by the animacy of the focal word. Results are collapsed on list number. Error bars are one SEM.

We considered participants’ contiguity of recall by calculating lag conditional response probability (lag-CRP) curves for animate and inanimate items (Figure 2). For each item recalled (the “focal item”), we calculated the likelihood that the next item recalled was encoded up to five positions (lag) before or after that focal item during encoding (cf. Kahana, 1996; Howard and Kahana, 1999). Although some lags exceeded ±5 items, we limited presentation and analysis to these bounds because most items were within this range and it is the norm for reporting CRP curves. These curves were typical: dropping quickly as lag increased in absolute magnitude but favoring forward recall (especially at lag +1) over backward recall.

Probability of recall was greater around inanimate than around animate items, *F*(1,93) = 15.78, *MSE* < 0.01, *p* < 0.001, *η_p_*^2^ = 0.15, and also differed by lag, *F*(9,837) = 109.73, *MSE* < 0.01, *p* < 0.001, *η_p_*^2^ = 0.54. Contrasts revealed that the probability of recall at lag +1 was greater than at all other lags (all *ps* < 0.001). Similarly, the probability of recall at lag −1 was greater than at all other lags (*ps* < 0.001) besides lag +2 (*p* = 0.169) and was lower than the probability at lag +1 (*p* < 0.001). Animacy and lag interacted, *F*(9,837) = 3.54, *MSE* < 0.01, *p* < 0.001, *η_p_*^2^ = 0.04. Paired comparisons indicated that the probability of recall was greater around inanimate than around animate items at lag +1 (*p* = 0.001) and lag +4 (*p* = 0.017) but did not differ at the other lags, yielding an interaction. Upon conducting a Bonferroni correction for the 10 comparisons, only the difference at lag +1 would remain significant. Given our large sample size, these two differences could also reflect Type I error.

Although a typical pattern of contiguity was apparent in the present results, animate words did not show greater contiguity than inanimate words, despite being better remembered. This outcome further supports the idea that the animacy advantage stems from item-specific processing but probably not from the greater temporal organization for animate over inanimate words (cf. Blunt and VanArsdall, 2021; but see Gelin et al., 2017).

## Discussion

People tend to remember more animate than inanimate concepts in free recall (e.g., Nairne et al., 2013, 2017; VanArsdall et al., 2013, 2017; Leding, 2018, 2019), but we do not know what produces this advantage mechanistically. We compared the retrieval dynamics of animate and inanimate words in a typical free recall task (with no buffer words and no distracter task between study and recall) to better understand the nature of this effect. Participants were more likely to recall animate than inanimate words, indicating the animacy advantage, but we found few, if any, differences in retrieval dynamics for animate vs. inanimate words. The animacy advantage was consistent across serial position in the lists, occurring in both the primacy and recency regions (regardless of their operationalization), as well as in the other regions of the lists. Participants did not favor animate over inanimate words in terms of probability of first recall or overall output order, and they also did not demonstrate clustering by animacy at retrieval. Animate words did not demonstrate greater retrieval contiguity than inanimate words (in fact, there was some evidence that *inanimate* words showed greater contiguity). Overall, the present results suggest that the animacy advantage stems from increased item-specific memory processing for animate over inanimate words (cf. Blunt and VanArsdall, 2021). The effect seems unlikely to stem from intentional differences in encoding or retrieval by animacy or categorical strategies (cf. VanArsdall et al., 2017).

Although the present results do not identify the proximate mechanism(s) that produces the animacy advantage, they can be useful for considering whether a proposed mechanism is feasible or not. We suggest that future tests of proximate mechanisms for the animacy advantage consider the implications of candidate mechanisms not just for overall recall, but for retrieval dynamics as well. These could be descriptive as in the present study, or studies could take advantage of methods known to reduce or enhance specific aspects of retrieval dynamics and purposely leverage them in tests of a given mechanism. Considering retrieval dynamics in this context can help to inspire, constrain, and test new accounts for the animacy advantage.

## Data Availability Statement

The datasets presented in this study can be found in online repositories. The names of the repository/repositories and accession number(s) can be found at: OSF, https://osf.io/rfbc2/.

## Ethics Statement

The studies involving human participants were reviewed and approved by Human Research Protection Program, Texas Tech University. The patients/participants provided their written informed consent to participate in this study.

## Author Contributions

The author confirms being the sole contributor of this work and has approved it for publication.

### Conflict of Interest

The author declares that the research was conducted in the absence of any commercial or financial relationships that could be construed as a potential conflict of interest.
